# Amplitude synchronization of spontaneous activity of medial and lateral temporal gyri reveals altered thalamic connectivity in patients with temporal lobe epilepsy

**DOI:** 10.1038/s41598-022-23297-4

**Published:** 2022-11-01

**Authors:** Anish V. Sathe, Michael Kogan, KiChang Kang, Jingya Miao, Mashaal Syed, Isaiah Ailes, Caio M. Matias, Devon Middleton, Feroze B. Mohamed, Scott Faro, Joseph Tracy, Ashwini Sharan, Mahdi Alizadeh

**Affiliations:** 1 Department of Neurosurgery, Thomas Jefferson University, Philadelphia, USA; 2 Department of Neurosurgery, University of New Mexico, Albuquerque, USA; 3 Department of Radiology, Thomas Jefferson University, Philadelphia, USA; 4 Department of Neurology, Thomas Jefferson University, Philadelphia, USA

**Keywords:** Epilepsy, Translational research, Predictive markers

## Abstract

In this study, we examined whether amplitude synchronization of medial (MTL) and lateral (LTL) temporal lobes can detect unique alterations in patients with MTL epilepsy (mTLE) with mesial temporal sclerosis (MTS). This was a retrospective study of preoperative resting-state fMRI (rsfMRI) data from 31 patients with mTLE with MTS (age 23–69) and 16 controls (age 21–35). fMRI data were preprocessed based on a multistep preprocessing pipeline and registered to a standard space. Using each subject’s T1-weighted scan, the MTL and LTL were automatically segmented, manually revised and then fit to a standard space using a symmetric normalization registration algorithm. Dual regression analysis was applied on preprocessed rsfMRI data to detect amplitude synchronization of medial and lateral temporal segments with the rest of the brain. We calculated the overlapped volume ratio of synchronized voxels within specific target regions including the thalamus (total and bilateral). A general linear model was used with Bonferroni correction for covariates of epilepsy duration and age of patient at scan to statistically compare synchronization in patients with mTLE with MTS and controls, as well as with respect to whether patients remained seizure-free (SF) or not (NSF) after receiving epilepsy surgery. We found increased ipsilateral positive connectivity between the LTLs and the thalamus and contralateral negative connectivity between the MTLs and the thalamus in patients with mTLE with MTS compared to controls. We also found increased asymmetry of functional connectivity between temporal lobe subregions and the thalamus in patients with mTLE with MTS, with increased positive connectivity between the LTL and the lesional-side thalamus as well as increased negative connectivity between the MTL and the nonlesional-side thalamus. This asymmetry was also seen in NSF patients but was not seen in SF patients and controls. Amplitude synchronization was an effective method to detect functional connectivity alterations in patients with mTLE with MTS. Patients with mTLE with MTS overall showed increased temporal-thalamic connectivity. There was increased functional involvement of the thalamus in MTS, underscoring its role in seizure spread. Increased functional thalamic asymmetry patterns in NSF patients may have a potential role in prognosticating patient response to surgery. Elucidating regions with altered functional connectivity to temporal regions can improve understanding of the involvement of different regions in the disease to potentially target for intervention or use for prognosis for surgery. Future studies are needed to examine the effectiveness of using patient-specific abnormalities in patterns to predict surgical outcome.

## Introduction

Epilepsy is a disease of abnormal neuronal connections between different regions of the brain^1^. Around 60–70% of patients with epilepsy are able to control their seizures via pharmacological treatment^2,3^. However, in a significant minority of patients with epilepsy, seizures remain refractory to medical management. Temporal lobe epilepsy (TLE) is the most common form of refractory focal epilepsy^4,5^. Mesial temporal sclerosis (MTS) is a form of mesial TLE (mTLE) and can cause severe complications including memory deficits and cognitive impairment^6^. Estimates of the prevalence and incidence of medically refractory mTLE with MTS in the U.S. range from 0.51 to 0.66 cases per 1000 people and 3.1–3.4 cases per 100,000 people per year, respectively, showing a significant burden of the disease^7^.

TLE is the most common type of epilepsy referred for epilepsy surgery^8^. Surgical management is the current treatment of choice for refractory epilepsy and is often effective at preventing recurrent seizures in patients^9^. However, while effective in epilepsy management, surgery has associated risks, including surgical, neurologic, and psychiatric complications^10–12^. Methods to identify areas for resection are still changing and improving^13^. Even more, while effective, surgical intervention may not lead to complete seizure freedom in MTS patients. Clinical and demographic data have been analyzed in the literature to uncover differences between seizure-free (SF) and not-seizure-free (NSF) populations and potentially prognosticate response to surgery. Variables including older age, increased duration of epilepsy, and presence of tonic–clonic seizures are associated with worse outcomes^14,15^. Analysis using models of functional connectivity in TLE patients has been shown to be capable of separating SF from NSF patients, indicating a difference in underlying pathologic connections may be associated with patient response to surgery^16^.

Surgical techniques to operatively manage MTS have also been changing. While anterior temporal lobectomy (ATL), an open procedure, is the most widely used surgical technique to treat MTS, the minimally invasive technique of laser interstitial thermal therapy (LITT) is becoming increasingly more common^17^. LITT has shown similar efficacy to ATL along with lower costs and rates of complications^17,18^.

There is currently established literature comparing specific connectivity patterns of MTS patients with those of healthy controls using functional connectivity analysis in resting-state fMRI (rsfMRI)^19–22^. Prior studies such as He et al. (2017) have examined functional connectivity patterns through network analysis and found thalamic hubness to be an important predictor of postsurgical seizure outcomes in patients who received ATL^23^.

However, to the best of our knowledge, there is a lack of studies that have used the technique to relate connectivity patterns with SF status in patients with refractory mTLE with MTS who received LITT rather than ATL. Elucidating noninvasive imaging biomarkers for abnormal epileptic connections could help identify different subtypes of MTS, aid in prognosis for response to LITT, and better determine areas involved in seizure networks to target for surgical intervention^13^. Such subclassification of MTS may reveal pathophysiological information that could be used to improve surgical outcomes for patients^24^. Importantly, there is also limited literature examining differences in functional connectivity patterns in MTS patients based on temporal lobe sub-segmentation into mesial (MTL) and lateral (LTL) regions^25,26^. This method of segmentation is relevant to MTS pathogenesis and surgical treatment and therefore warrants investigation^27,28^.

In this study, we utilize patient rsfMRI data to identify differences in functional connectivity between temporal lobe regions and other brain regions in patients with medically refractory mTLE with MTS and healthy controls. Uniquely, we examine temporal lobe connectivity based on its sub-segmentation into mesial and lateral sections, providing more insight into connectivity differences in regions relevant to MTS pathogenesis, anatomy, and surgery. We further analyze connectivity differences between patients who remained SF for 12 months after receiving LITT and those who did not. Through this analysis, we expect to find differences in temporal lobe functional connectivity to other brain regions, specifically the thalamus, in patients with mTLE with MTS based on seizure status after LITT. We aim to determine the degree and locations of altered functional connectivity with the MTL and LTL to potentially better inform surgical site selection. We also look at whether we can predict patient response to LITT based on noninvasive, presurgical imaging.

## Methods

### Subject demographics

We analyzed data from 25 patients with left-sided MTS (age 23–69, median age 52), 6 patients with right-sided MTS (age 22–67, median age 50), and 16 healthy controls (age 21–35, median age 23). 2 patients with right-sided MTS were SF and 4 were NSF at 1 year post-op, while 11 patients with left-sided MTS were SF and 14 were NSF at 1 year post-surgical follow up. Information about subject characteristics is included in Table 1. All patients with mTLE with MTS included in this study received laser interstitial thermal therapy (LITT) as surgical intervention. Patient pre-operative resting-state fMRI scans were used for image analysis. All healthy controls were recruited for the purposes of this study and were neurologically intact as participants. This study was approved by the institutional review board (IRB) of Thomas Jefferson University Hospital. All methods were performed in accordance with the relevant guidelines and regulations approved by IRB. Informed consent was obtained from all subjects included in the study. The clinical diagnosis of mTLE was made according to the criteria of the International League Against Epilepsy (ILAE)^29^. These patients underwent preoperative evaluation consisting of history, semiology, neuropsychological evaluation, video electroencephalography, and sometimes intracranial electroencephalography, 18F-FDG PET, and anatomic and functional MRI^30^. These modalities helped make the diagnosis, localize the epileptogenic focus, and assess for application of LITT.

**Table 1:** Subject characterestics.

Group	Number of subjects	Median age at scan	Age range	Median duration of epilepsy	Left-sided focus of MTS	% Left-sided focus
Controls	16	23	21–35	N/A	N/A	N/A
MTS total	31	52	22–69	23	25	81%
MTS SF	13	53	31–67	27	11	85%
MTS NSF	18	50	22–69	19.5	14	78%

### Image acquisition

All patients underwent rsfMRI scans prior to surgery using a 3.0 T Phillips scanner with an eight-channel head coil. rsfMRI images were acquired axially using a single-shot echo planar imaging (EPI) sequence in the same anatomical location prescribed for T1-weighted 3D MPRAGE images. The T1-weighted imaging parameters used were: FOV = 24.0 cm, voxel size = 1.0 × 1.0 × 1.0mm3, matrix size = 512 × 512, TR = 12 ms, TE = 6 ms and slice thickness = 1 mm. Resting state imaging parameters were FOV = 23.0 cm, voxel size = 3.0 × 3.0 × 3.0 mm3, matrix size = 128 × 128, TR = 2.5 s, TE = 62 ms, number of averages = 1 and acquisition time = 12 min (360 volumes). Participants were instructed to relax, keep their eyes open and think of nothing in particular during the resting state scan.

### Image processing

All the subject images were processed through a conventional, established preprocessing pipeline using the FSL MELODIC toolbox^31^. In this pipeline, subject rsfMRI scans underwent MCFLIRT motion correction, skull stripping, smoothing, and normalization. A high-pass filter was also applied, with 100 s designated as the longest period of time which would be included. Next, these processed functional scans were registered via linear registration to the high-resolution structural T1-weighted image and then subsequently to the Montreal Neurological Institute (MNI) standard space using a resampling resolution of 2 mm. Output data from each subject consisted of filtered_func_data, which was the filtered functional data over a time course, as well as mean_func, which was an intensity-based image with each voxel holding a value averaged across the time domain.

Subject T1-weighted structural MRI scans were automatically segmented using Freesurfer software (version 7.1.1) utilizing Desikan-Killiany parcellation into left and right MTL and LTL sub-segmentations^32,33^. These automatic segmentations were manually corrected as necessary. These four sub-segmentations were used as seeds for further analysis.

Patient T1-weighted images were registered to mean_func using quick symmetric normalization registration followed by symmetric normalization from the ANTs toolbox^34^. The transformation matrices generated from the registrations were applied to each of the left and right MTL and LTL segmentations obtained from each patient.

### Functional connectivity analysis

We utilized seed-to-voxel analysis via amplitude synchronization of BOLD signal fluctuations to measure functional connectivity^35^. Dual regression in the time and space domains was done on the filtered_func functional image in the regions designated by the registered segmentations to detect correlations. This calculated synchronization of signal amplitude in filtered_func from the four temporal lobe sub-segmentation seeds to each voxel in the brain^36^. The amplitude synchronization represents the correlations in the amplitudes of the waveforms of the BOLD signal fluctuations in the seed regions to each voxel over the whole time course of the functional image. This analysis provided outputs in the form of statistical maps populated with Z-scores representing the level of correlation of the amplitude synchronization from each seed to each voxel in the brain.

Brain regions were automatically segmented from the subject T1-weighted scans, including the thalamus (whole and bilateral), insula (bilateral), cerebellum (bilateral), brainstem, total gray matter, medial and lateral temporal gyri (bilateral) and whole supratentorial region (bilateral). The selected regions were based on evidence-driven hypotheses with knowledge of involvement of these regions in TLE^37–40^. These region masks were registered to the subject averaged functional scans (mean_func) using the same transformation matrices used to register the temporal lobe sub-segmentations. Next, we calculated volume overlap of significantly synchronized voxels in each region using each seed, doing the same for each subject. These data were reported as the proportion of significant voxels in the whole region volume. These values were generated for both positively and negatively correlated amplitudes. Positive correlations represent stimulation between the regions while negative correlations indicate inhibition. For controls, correlations were analyzed based on ipsilateral (left-to-left and right-to-right) and contralateral (left-to-right and right-to-left) connections. Both sets of ipsilateral correlations in the controls (left-to-left and right-to-right) were used to compare to ipsilateral correlations in patients with mTLE with MTS, and the same for contralateral correlations. Each control was therefore used twice in ipsilateral vs contralateral analysis. Brain regions of patients with mTLE with MTS were categorized based on laterality compared to the lesional side. Control ipsilateral and contralateral connections were directly compared. However, structures located in the midline, such as the brainstem, were unable to be analyzed this way. MTS, SF and NSF patient correlations were compared directly with analogous connections in controls. Heatmaps generated from correlation data were made using the Data Processing and Analysis for (Resting-state) Brain Imaging (DPABI) software tool^41^.

### Statistical analysis

The proportional volume overlap data from subjects was analyzed using a general linear model and compared with homogeneity tests based on construction of a 95% confidence interval. The variables of seizure duration and age at MRI acquisition were used as covariates in the model. Bonferroni correction was used to correct for multiple comparisons. Means were compared using estimated marginal means. Significant differences in both positive and negative correlations between ipsilateral and contralateral control connections; controls and patients with mTLE with MTS; and controls, SF patients with mTLE with MTS, and NSF patients with mTLE with MTS were reported. Data from patients with left-sided MTS and right-sided MTS were analyzed together and correlations to significant regions were calculated as ipsilateral or contralateral to the seizure focus. Structures ipsilateral to the seizure focus were called lesional-side (LS), while contralateral structures were termed nonlesional-side (NLS). Significant differences were reported for values of p < 0.05.

## Results

### Temporal lobe connectivity in healthy controls

**Figure 1:**
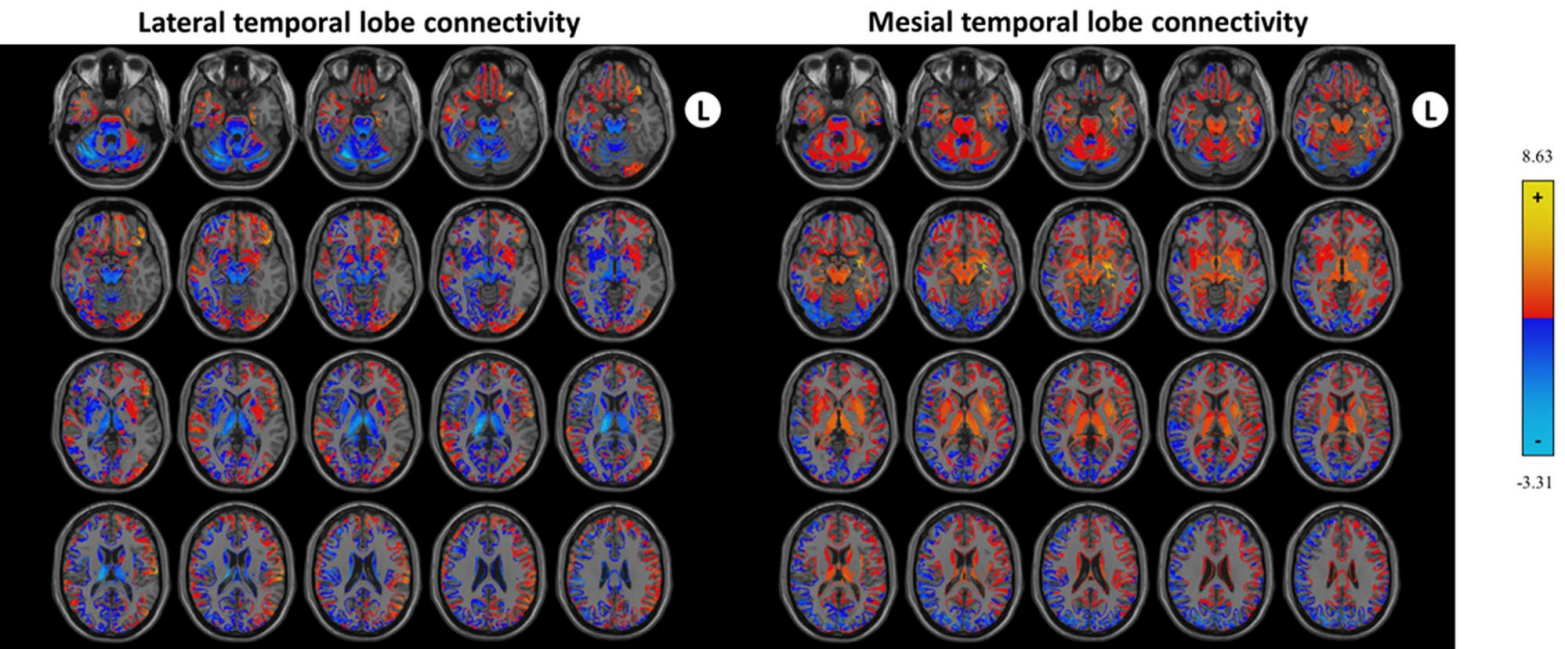
Temporal lobe connectivity from the lateral and MTLs in controls. Connectivity patterns with the rest of the brain are shown from the LTL (left) and the MTL (right).

**Figure 2:**
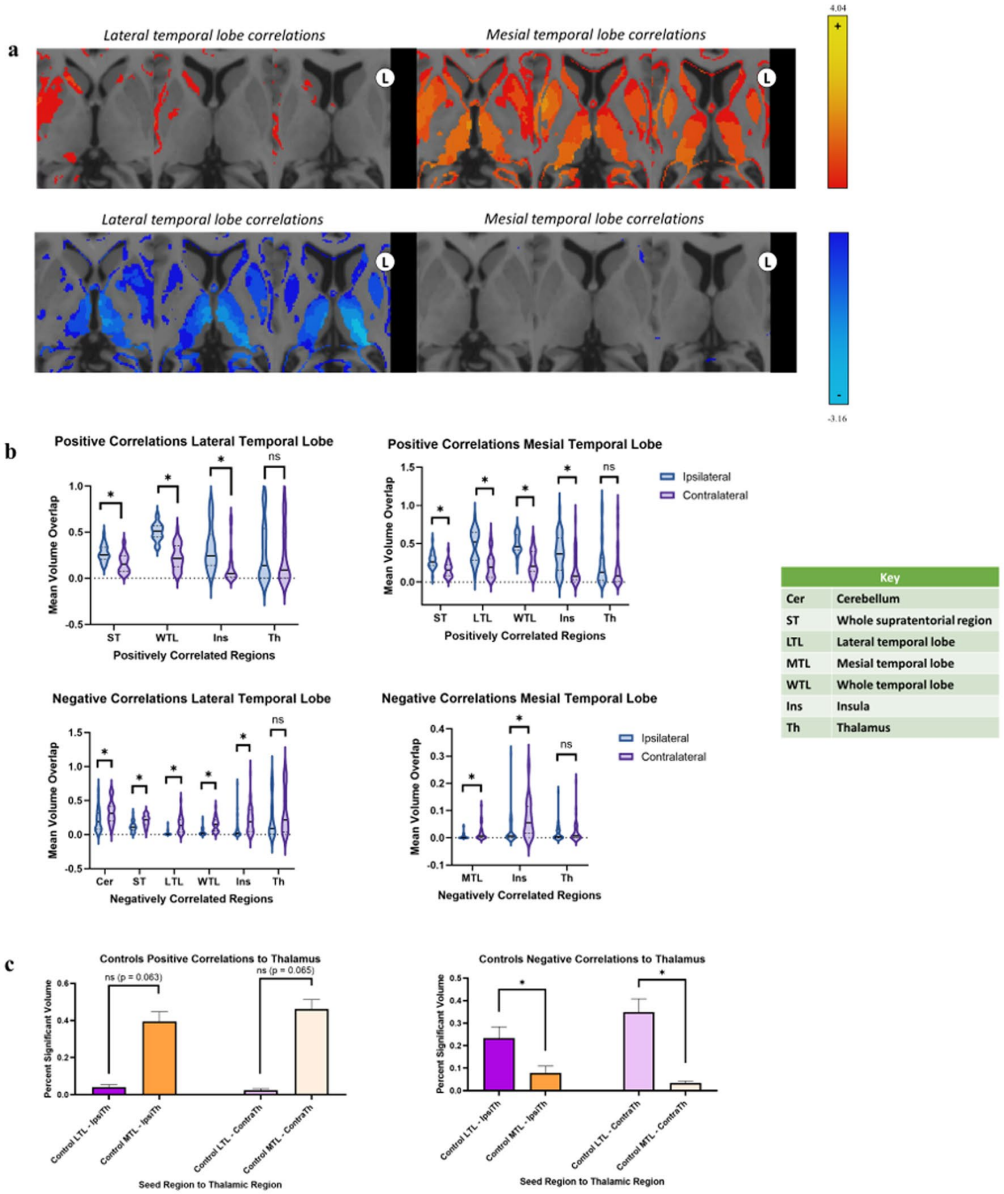
Positive and negative temporal lobe connectivity in healthy controls. (**a**) Heatmaps of connectivity from the LTL (left) and MTL (right) to thalamic regions. (**b**) Comparisons of positive and negative connectivity between ipsilateral and contralateral connections with the seed as the LTL (left) and the MTL (right). (**c**) Comparisons of positive and negative connectivity between the LTL and Th and the MTL and Th. In all sections, regions with significant differences found at p < 0.05 are marked with *. Regions without significant differences are marked with “ns” for not significant. Images are displayed in radiographic orientation. Correlations shown are based on ipsilateral and contralateral correlations from the temporal lobe; as such, the right-side temporal lobe seed heatmaps were mirrored to combine with the left-side seed heatmaps for visualization purposes. The left and right sides of the images are marked with L and R; the side that was used as the seed is the one displayed on the image.

Connectivity from the mesial and lateral temporal lobes was measured in healthy controls. A heatmap of the average connectivity from all of the controls is displayed in Fig. 1. Positive and negative correlations with the thalamus are represented in Fig. 2a.

When directly compared, the MTLs in controls showed an increased proportion of positive voxels compared to negative to the total brain (p = 0.003) and other structures including the thalamus (p = 0.035) and ipsilateral insula (p < 0.001). Meanwhile, the LTLs did not show a difference between overall positive and negative connectivity with the total brain, but did show higher proportions of positive correlations with structures such as the ipsilateral temporal lobe (p < 0.001) and insula (p < 0.001).

Structures that had significant differences in temporal lobe connectivity between ipsilateral and contralateral sides included the whole supratentorial regions, the temporal lobes (segmented and whole), and the insula (p < 0.001). The thalamus did not show significant differences in temporal lobe connectivity based on laterality. In these healthy controls, the LTL had higher ipsilateral than contralateral connections to numerous regions for positive correlations as depicted in Fig. 2b, with similarly higher negative correlations to the same contralateral structures compared to ipsilateral in the whole supratentorial regions. However, correlations to more midline structures such as the thalamus appeared symmetric in these individuals.

Normal controls also showed differences in connectivity from structures to the mesial and LTLs. Structures such as the thalamus (ipsilateral, contralateral and whole), brainstem, contralateral cerebellum, contralateral MTL, and the contralateral insula showed lower proportions of negative correlations to the MTL compared to the LTL. Notably, the LTLs showed higher negative correlations than the MTLs to the thalamus, both ipsilateral (p = 0.006) and contralateral (p < 0.001), as seen in Fig. 2c. A similar inversion of connectivity between positive and negative correlations was ultimately noted with mesial temporal structures in healthy controls, with mainly the insula showing larger proportions of positive ipsilateral correlations (Fig. 2b, p < 0.001) and negative contralateral correlations (Fig. 2b, p = 0.01). Thalamic connectivity was noted to have no significant asymmetry to the mesial temporal region (p = 0.536). As shown in Fig. 2c, the MTLs did not show significantly higher positive correlations than the LTLs to the thalamus, although these were edge cases (ipsilateral thalamus p = 0.063, contralateral thalamus p = 0.065).

### Temporal lobe connectivity differences between patients with mTLE with MTS and controls

**Figure 3:**
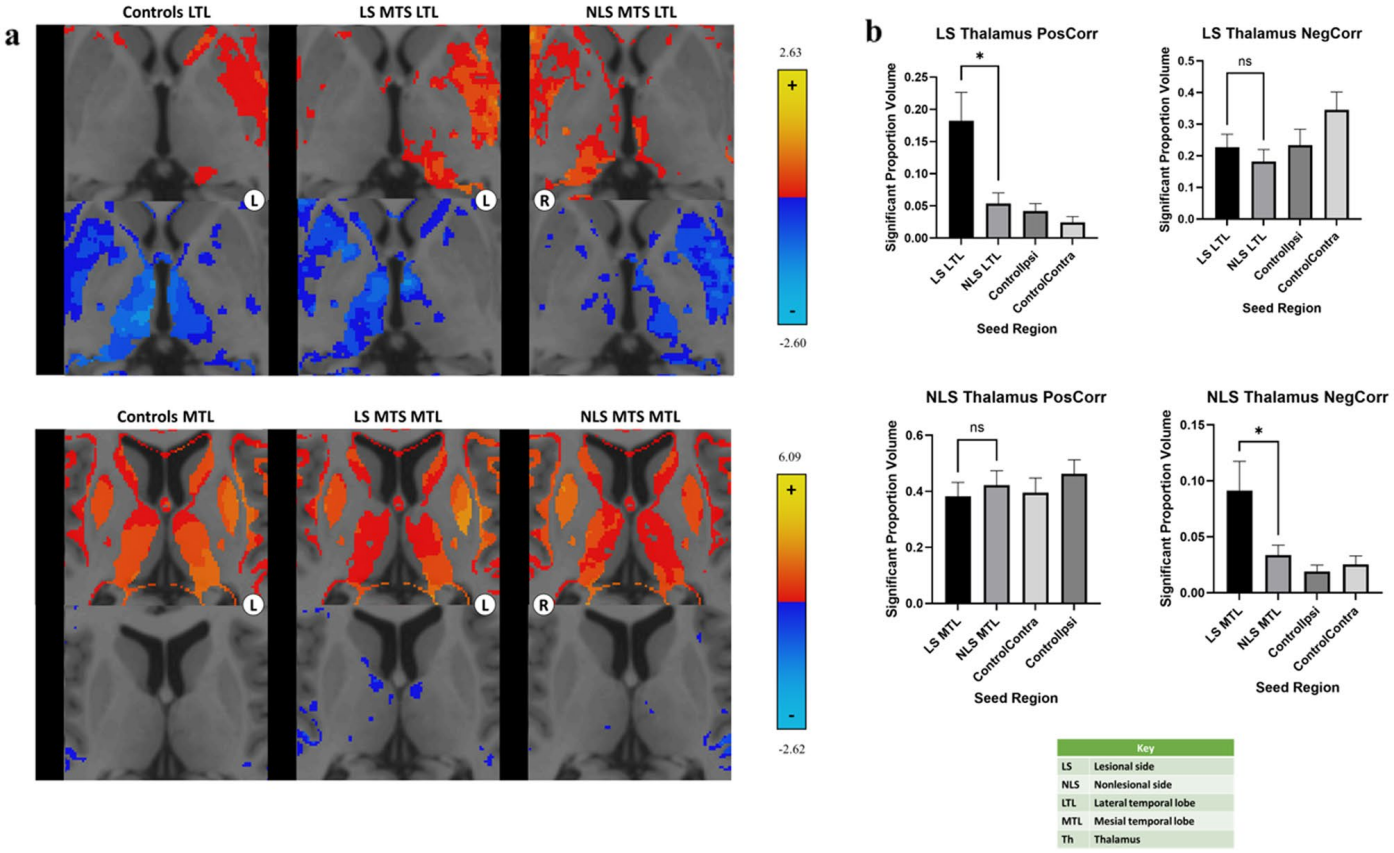
LTL and MTL connectivity to the thalamus. (**a**) Heatmaps of correlations from the LTL (top) and MTL (bottom) to the thalamus are shown. Heatmaps of patients with mTLE with MTS with a right-sided focus were mirrored so that the focus of all patients was on the anatomical left side for visualization. These were combined with heatmaps from patients with a left-sided MTS focus. Regions with z > 2.5 or z < − 2.5 are shown. Images are displayed in radiographic orientation. The anatomic left and right sides of the images are marked with L and R; the side that was chosen as the seed is the one marked on the image. (**b**) Comparisons between connectivity from the LS and NLS LTL (top) and MTL (bottom) to the thalamus are shown based on laterality of connections. ControlIpsi shows connectivity from the temporal lobe seed to the ipsilateral thalamus; ControlContra shows connectivity from the temporal lobe seed to the contralateral thalamus. Correlations with significant differences are marked in the graphs with *. Selected nonsignificant differences are marked with “ns” for nonsignificant.

We found that the LS LTL showed a higher proportion of positive correlations to the LS thalamus in patients with mTLE with MTS compared to controls (p = 0.031), but similar levels of negative correlations. Conversely, the LS MTL showed similar levels of both positive and negative correlations to the NLS thalamus compared to controls. Overall, both controls and patients with mTLE with MTS showed higher positive correlations with the thalamus from the MTL compared to the LTL, and higher negative correlations with the LTL compared to the MTL. Heatmaps depicting correlations from the LTL and MTL are shown in Fig. 3a.

Our findings also show differences in correlations to the thalamus based on laterality when comparing patients with mTLE with MTS to controls. Looking at correlations with the LS thalamus, the LS LTL has significantly higher positive correlations compared to the NLS LTL in patients with mTLE with MTS (p = 0.002) but no significant difference in controls (p = 1.000), as seen in Fig. 3b. There is no significant difference in negative correlations to the LS thalamus or any correlations to the NLS thalamus based on laterality from the LTLs in patients with mTLE with MTS and controls.

Meanwhile, when looking at correlations with the NLS thalamus, the LS MTL shows higher negative correlations compared to the NLS MTL in patients with mTLE with MTS (p = 0.043), as shown in Fig. 3b. This difference is not seen in controls (p = 1.000). There is no significant difference in positive correlations to the NLS thalamus or any correlations to the LS thalamus based on laterality from the MTLs in patients with mTLE with MTS and controls.

### Temporal lobe connectivity differences based on seizure outcomes

**Figure 4:**
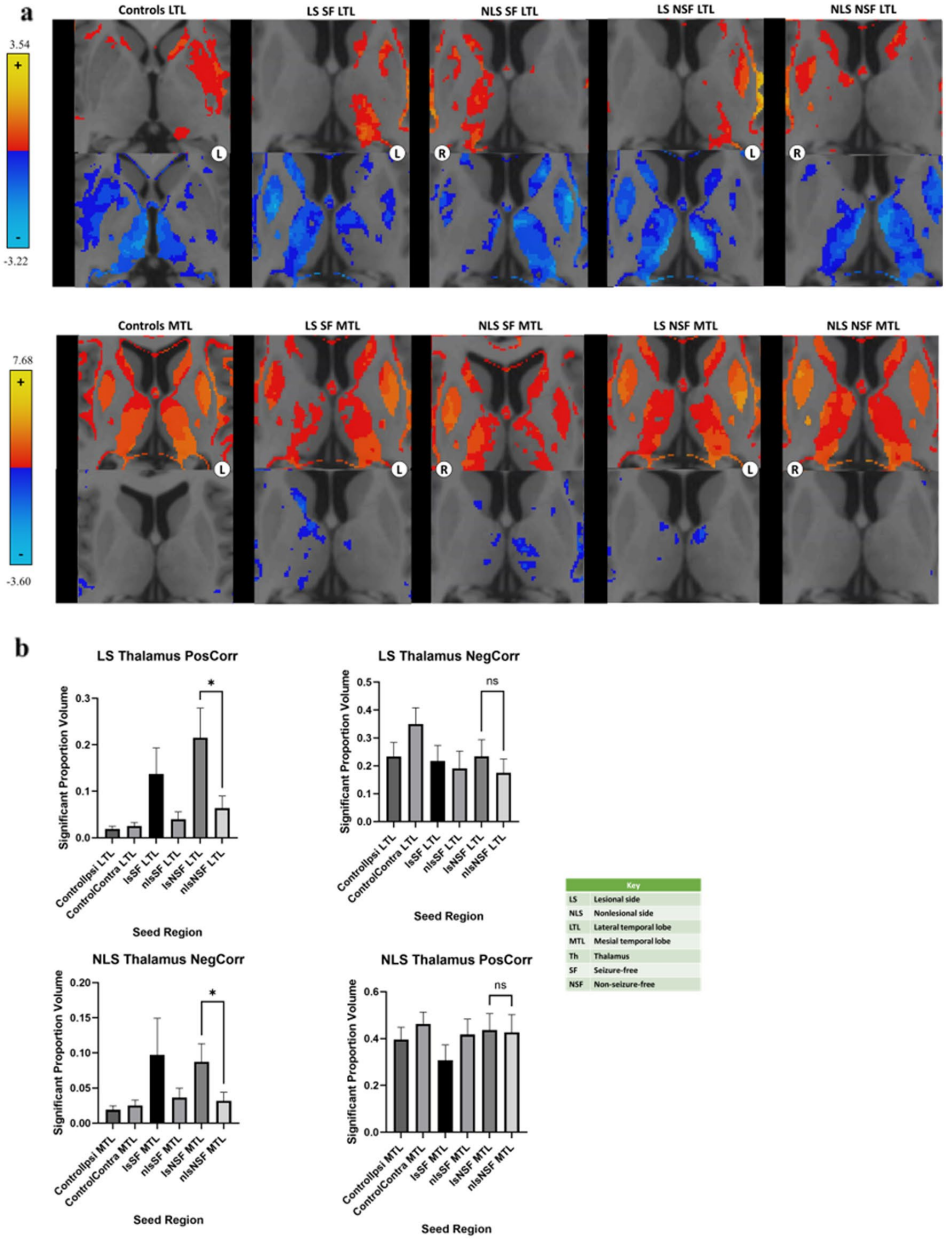
Combined heatmaps from the LTL and MTL of SF and NSF MTS patients. (**a**) Heatmaps of correlations from the LTL (top) and MTL (bottom) to the thalamus are shown. Heatmaps of patients with mTLE with MTS with a right-sided focus were mirrored so that the focus of all patients was on the anatomical left side for visualization. These were combined with heatmaps from patients with a left-sided MTS focus. Regions with z > 2.5 or z < − 2.5 are shown. Images are displayed in radiographic orientation. The anatomic left and right sides of the images are marked with L and R; the side that was chosen as the seed is the one marked on the image. (**b**) Comparisons between connectivity from the LS and NLS LTL (top) and MTL (bottom) to the thalamus are shown based on laterality of connections. ControlIpsi shows connectivity from the temporal lobe seed to the ipsilateral thalamus; ControlContra shows connectivity from the temporal lobe seed to the contralateral thalamus. Correlations with significant differences are marked in the graphs with *. Selected nonsignificant differences are marked with “ns” for nonsignificant.

Differences in correlations were seen between temporal lobe segments and brain regions depending on the clinical outcomes of patients with mTLE with MTS. These differences were based on laterality compared to the lesional side. We found that the LS LTL showed higher positive correlations with the LS thalamus compared to the NLS LTL in NSF patients (Fig. 4b, p = 0.018). However, this asymmetry was not seen when examining SF patients and controls (p = 1.000). Furthermore, no such asymmetry was seen when looking at negative correlations with the LTL (p = 1.000). Meanwhile, the LS MTL showed higher negative correlations with the NLS thalamus compared to the NLS MTL in NSF patients (Fig. 4b). However, this comparison was only significant in the general linear model before Bonferroni correction was applied. In contrast, SF patients and controls retained relatively symmetric negative correlations (p = 1.000). No asymmetry was seen when examining positive correlations with the MTL (p = 0.251). Heatmaps depicting correlations based on seizure outcomes are seen from the LTL and MTL in Fig. 4a.

## Discussion

### Healthy controls show differences in connectivity based on laterality

Functional connectivity analysis in healthy controls revealed higher overall positive correlations between the temporal lobes and ipsilateral structures and higher proportions of negative correlations between the temporal lobes and contralateral structures. Regions with significant differences mainly consisted of cortical and cerebellar areas, including the temporal lobe and insula. Thalamic structures did not differ in connectivity based on laterality, indicating relatively equal, bilateral functional connectivity at baseline in healthy brains.

Prior literature has shown higher intra-temporal lobe connectivity measures in the left temporal lobe compared to the right in healthy controls, with some connections increased in the right compared to the left^42^. However, as we combined left-sided and right-sided correlations to focus on ipsilateral and contralateral connections, these differences should not impact our analysis.

### Patients with mTLE with MTS show differences in connectivity compared to controls

Our analysis revealed significant differences in functional connectivity in patients with mTLE with MTS compared to healthy controls. Namely, the LS LTL shows increased positive correlations with the LS thalamus, underscoring involvement of this region in seizure propagation networks. The thalamus is known to be involved in seizure spread and alterations in thalamic connectivity have been studied in different forms of epilepsy^43^.

While the thalamus in healthy controls showed relative symmetry in its connections with the bilateral temporal lobes, the thalamus in patients with mTLE with MTS shows loss of this symmetry. The LS LTL shows higher positive correlations with the LS thalamus compared to the NLS temporal lobe, while the LS MTL shows higher negative correlations with the NLS thalamus compared to the NLS temporal lobe. Haneef et al.^44^ also found increases in functional connectivity between the hippocampus and the thalamus in patients with TLE^44^. Golden et al.^46^ and Jo et al. (2019) discuss increases seen in epilepsy patients in functional connectivity between cortical regions and the mediodorsal nucleus of the thalamus, a nucleus known to be involved in seizure propagation^45,46^.

These findings suggest an overall disruption of thalamic connectivity with temporal lobe regions and show a relative asymmetry between the LS and NLS temporal lobes being associated with abnormal seizure networks. The increases in positive and negative correlations between the thalamus and the LS temporal lobe could signify increased propensity for seizure propagation.

Englot et al.^16^ demonstrated lower functional connectivity between the ascending reticular activating system (ARAS) and cortical structures like the temporal lobe in TLE^47^. This system is anatomically based throughout the brainstem and is involved in arousal and maintenance of consciousness^48,49^. Involvement of this system in seizures may lead to the decrease in awareness and consciousness seen in certain seizure subtypes^47^. Regions composing this system include the brainstem and connections to components of the thalamus^50^. On analysis of MTS vs controls, the LS LTL shows increased positive connectivity to thalamic regions but not the brainstem. This suggests that, while the overall temporal lobe may show subtly decreased connections to the ARAS, the LTL may be hyperactive in its communication with the system.

Morgan et al. (2012) found differences in hippocampal connectivity to structures such as the ventral lateral nucleus of the right thalamus in patients with a right-sided focus of TLE compared to a left-sided focus^51^. However, these results do not contradict our own as they similarly reflect differences in functional connectivity based on ipsilateral/contralateral temporal lobe-thalamic connections. Furthermore, other studies have found relatively similar functional alterations in patients with TLE regardless of left or right-sided seizure focus^44^. Our analysis accounted for potential differences in left- and right-sided pathology by grouping regions based on whether they were ipsilateral or contralateral to the affected side and combining left and right-sided focus patients together. As such, our results demonstrate alterations in temporal lobe connectivity in MTS that occur relative to the focus of the disease rather than strictly in the left or right hemisphere.

### Pre-operative functional connectivity of patients with mTLE with MTS differs based on clinical outcomes

Analysis of patients based on SF status at 12 months post-surgery revealed differences in functional connectivity between groups. Both SF and NSF patients showed increases in positive correlations between the LS LTL and the LS thalamus compared to controls, indicating that thalamic hyperconnectivity could increase seizure propagation in abnormal epileptogenic networks. Higher thalamic activity leading to greater whole-brain network integration has been implicated in nonresponse to surgery, although the nature of thalamic hyperactivity in that study was extratemporal and contralateral^23^.

DeSalvo et al.^52^ showed that more diffuse, less integrated connectivity and disruption in the NLS temporoinsular region may predict lack of seizure freedom after surgery^52^. In NSF patients, the NLS MTL showed decreased negative correlations with the NLS thalamus compared to the LS MTL, although this difference was an edge case and not significant (p = 0.059). Analysis with more patients may be able to discern a statistical difference.

We also found asymmetry in correlations between the bilateral temporal lobes and thalamus in NSF patients but not in SF patients or controls. This asymmetry represented increased correlations from the LS temporal lobe compared to the NLS temporal lobe, potentially revealing increases in abnormal thalamic connections leading to hyperactive seizure spread networks in poor responders to epilepsy surgery. Furthermore, the asymmetry shows both increases in negative correlations with the MTL and positive correlations with the LTL from the NLS and LS thalamus, respectively, although the MTL correlations were only significant in the general linear model before Bonferroni correction. This indicates whole-thalamus and temporal lobe reorganization in NSF patients, with temporal lobe connectivity alterations differing between the mesial and lateral subsections of the region. These findings suggest that temporal lobe asymmetry may be prognostic for worse response to surgery and may reflect greater temporal-thalamic functional disorganization. Thom et al. (2015) suggested that involvement of extratemporal pathology may be one factor underlying nonresponse to epilepsy surgery^24^. The thalamic asymmetry seen in NSF patients may reflect a more widespread disruption of functional networks in NSF patients. To note, the SF patients did show some asymmetry with increased LS connections compared to NLS, but these differences were far from reaching statistical significance, possibly due to the higher variability seen in the SF cohort. These results still establish that NSF patients show consistent thalamic asymmetry while SF patients do not.

### Study limitations

The subject cohort used in this study contains a highly select group of patients with focal epilepsy with MTS. These results may not be able to be applied to patients with TLE but without MTS on MRI or confirmed by anatomo-pathological analysis. Additionally, epilepsy patients are not a homogenous group when examining disease characteristics such as focus location, trialed medications, extent of seizure spread, and bilateral MTS. The patients included in this study all had refractory TLE but were not grouped based on seizure type (e.g. focal aware vs focal with impaired awareness vs focal-to-tonic clonic). These variations in disease extent and clinical presentation may reflect differences in the pathophysiology underlying the disease in these patients which may influence the temporal lobe connectivity we examined. Future studies may utilize subject samples with a large enough sample size to group patients according to these different seizure classifications. Furthermore, these results were based on group analysis of patients with mTLE with MTS. Future studies analyzing patients individually may better establish the clinical utility of these findings.

Our imaging parameters included a sampling rate of 2.5 s as well as a voxel size of 3 × 3 × 3 mm^3^. Improvements in the temporal and spatial resolution of the functional imaging for these patients could generate more reliable data. Furthermore, while we found many alterations in functional connectivity involving the thalamus in this study, we did not have the necessary resolution to perform functional analysis on different sub-nuclei of the thalami. As each nucleus is involved in unique neurological functions, future studies with better sampling resolution may examine thalamic functional connectivity based on sub-segmentation into its various nuclei.

This study used only preoperative imaging from patients with mTLE with MTS, with clinical correlations made with seizure frequency post-LITT. Future studies could look at both preoperative and follow-up postoperative imaging to directly correlate functional connectivity alterations due to LITT with clinical outcomes. Additionally, while we focused on temporal lobe connectivity, especially with the thalamus, future studies could examine connectivity between different brain regions in patients who received LITT.

## Conclusion

Our analysis of temporal lobe functional connectivity via amplitude synchronization found that healthy controls show symmetric bilateral temporal lobe connectivity with thalamic structures, but differ based on laterality to supratentorial structures with both MTL and LTL regions. When compared to these baseline findings, patients with mTLE with MTS are characterized by an increase in asymmetric connectivity to thalamic areas. This asymmetry is present when looking both at the MTL and LTL; however, the LTL shows asymmetry in positive correlations while the MTL shows asymmetry in negative correlations.

The deviations from baseline control connectivity were seen both when patients with mTLE with MTS were compared to controls as a whole and when these patients were split based on SF status at 1 year, indicating the existence of underlying pathology leading to alterations in brain connectivity that may lead to the formation of abnormal seizure networks in MTS. We found significant differences in temporal lobe thalamic connectivity between SF and NSF patients with mTLE with MTS and with controls, suggesting alterations in functional connectivity may be a driver or a prognostic factor of SF outcomes after surgery for MTS. Our analysis also suggests increased asymmetry in thalamic connectivity predicts a reduced response to mesial temporal ablation, while bilateral thalamic connections with the temporal lobe predicts a better response. Notably, these parameters can be examined using data contained within a single patient’s fMRI by comparing LS and NLS temporal-thalamic connectivity, putting forth thalamic asymmetry as a noninvasive, presurgical biomarker that could be used to determine candidates who will be responsive to epilepsy surgery.

Our work also uniquely split our examination of connectivity of the temporal lobe into mesial and lateral sections, providing analysis between regions relevant to surgical intervention. This analysis may better provide insight into deciding which temporal lobe connections are altered in patients with mTLE with MTS to improve understanding of the pathology of the disease, find involvement of different brain regions, and potentially assist in planning for surgery.

## Supplementary Information

Supplementary Information.

## Data Availability

All raw data generated for analysis for the purposes of this study are included in this article and its associated Supplementary Files.
